# Semen cryopreservation, utilisation and reproductive outcome in men treated for Hodgkin's disease

**DOI:** 10.1038/sj.bjc.6600483

**Published:** 2002-08-12

**Authors:** F H Blackhall, A D Atkinson, M B Maaya, W D J Ryder, G Horne, D R Brison, B A Lieberman, J A Radford

**Affiliations:** Cancer Research UK Department of Medical Oncology, Christie Hospital NHS Trust, Wilmslow Road, Manchester M20 4BX, UK; Department of Medical Statistics, Christie Hospital NHS Trust, Wilmslow Road, Manchester M20 4BX, UK; Subfertility Laboratory, Central Manchester and Manchester Childrens University Hospitals NHS Trust, Hathersage Road, Manchester M13 0JH, UK; Department of Reproductive Medicine, Central Manchester and Manchester Childrens University Hospitals NHS Trust, Hathersage Road, Manchester M13 0JH, UK

**Keywords:** semen cryopreservation, reproductive outcome, Hodgkin's disease

## Abstract

Between 1978 and 1990, 122 men underwent semen analysis before starting sterilising chemotherapy for Hodgkin's disease. Eighty-one (66%) had semen quality within the normal range, 25 were oligospermic (<20×10^6^ sperm per ml) and five were azoospermic (no sperm in the ejaculate). Semen from 115 men was cryopreserved and after a median follow-up time of 10.1 years, 33 men have utilised stored semen (actuarial rate 27%) and nine partners have become pregnant resulting in 11 live births and one termination for foetal malformation. Actuarial 10 year rates of destruction of semen before death or utilisation and death before utilisation are 19% and 13% respectively. This retrospective cohort study demonstrates that approximately one-quarter of men utilising cryopreserved semen after treatment for Hodgkin's disease obtain a live birth. The high non-utilisation rate is intriguing and warrants further investigation.

*British Journal of Cancer* (2002) **87**, 381–384. doi:10.1038/sj.bjc.6600483 www.bjcancer.com

© 2002 Cancer Research UK

## 

Most patients with Hodgkin's disease can expect prolonged survival due to the success of chemotherapy regimens which have evolved since the first introduction of MOPP (mustine, vincristine, procarbazine and prednisolone) in 1964 (de Vita *et al*, 1972). Unfortunately, infertility is a common late effect of treatment and particularly important since most patients are young adults at diagnosis (Whitehead *et al*, 1982). Semen cryopreservation is currently the only available technique to preserve reproductive potential in men undergoing potentially sterilising treatments and in the UK is offered routinely to patients who are under 55 years of age. However, little information exists as to the benefits of this policy. In this study, semen quality before and after chemotherapy, rates of utilisation of cryopreserved semen and reproductive outcome have been analysed in a cohort of 122 men who presented to a regional cancer centre with newly diagnosed Hodgkin's disease between 1978 and 1990.

## PATIENTS AND METHODS

Names of all male patients with newly diagnosed Hodgkin's disease registered at the Christie Hospital were cross-matched with the names of patients referred for semen cryopreservation at St Mary's Hospital, Manchester between January 1978 and December 1990. Permission to gather information from the files at St Mary's Hospital, Manchester was obtained from the Human Fertility and Embryology Authority. Details of patient age and stage of disease at diagnosis, treatment given, current disease status, dates and treatment of relapse, semen analysis before and after treatment, marital status and number of children fathered prior to diagnosis of Hodgkin's disease, use of cryopreserved semen, outcome of assisted reproduction and natural conceptions following treatment were obtained from case notes and the reproductive medicine data base. To compare semen quality between patients with multiple semen analyses a ‘best’ sample was selected by using a rank-sum procedure. Briefly, sperm concentration and motility were ranked independently and in ascending order, then the ranks of concentration and motility were summed for each sample. The highest rank-sum was used to select the ‘best’ semen specimen. The Kaplan–Meier method was used to estimate actuarial rates of semen utilisation, death without utilisation and destruction of semen without utilisation from the time of cryopreservation. The log-rank test was used to compare semen utilisation in men stratified according to marital status, number of children and age at diagnosis.

## RESULTS

**Figure 1:**
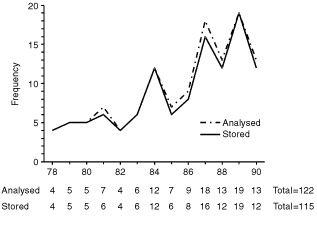
Men referred to Regional Fertility Centre between 1978 and 1990 for semen analysis and cryopreservation before treatment of Hodgkin's disease.

During the 12 year period 1978 to 1990, 122 men with a median age of 24 years (range 16–44) were eligible for inclusion in the study. As shown in Figure 1

, the annual rate of referrals made for semen cryopreservation increased over this time. All patients received MVPP (mustine, vinblastine, prednisolone, procarbazine) or ChlVPP/EVA hybrid (chlorambucil, vinblastine, prednisolone, procarbazine, etoposide, vincristine, doxorubicin) chemotherapy either as adjuvant treatment following radiotherapy in early stage disease (MVPP) or for advanced stage disease (MVPP or ChlVPP/EVA hybrid) followed by radiotherapy to sites of intial bulk or residual masses. These regimens have equivalent gonadal toxicity (Clark *et al*, 1995). In total, radiotherapy was administered to the pelvis following chemotherapy in three patients and to sites distant from the abdomen or pelvis in 82 patients.

### Semen analysis before treatment

A median of four semen samples was analysed for each patient (range 1–7). For 121 out of 122 patients with complete data, the median sperm concentration was 50×10^6^ per ml (range 0–306) and median sperm motility was 60 (range 0–90). Azoospermia (no sperm present in the ejaculate) and oligospermia (<20×10^6^ per ml) were present in five and 25 patients respectively and severe oligospermia (⩽10×10^6^ per ml) was present in 13 out of 25 patients.

**Figure 2:**
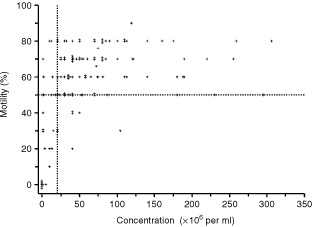
Sperm motility *vs* concentration in 121 men with Hodgkin's disease before start of treatment. Results for the ‘best’ samples analysed are shown (see Materials and Methods). Samples lying in the right upper quadrant, as defined by the horizontal line drawn at 50% motility and the vertical line drawn at 20 million per ml, are within the normal range for semen quality according to WHO criteria.

According to WHO criteria (Who Laboratory Manual, 1992) 66% of patients in this series had a semen quality within the normal range (sperm concentration >20×10^6^ per ml and >50% motile sperm) (Figure 2

). Semen was cryopreserved for 115 out of 122 patients (Figure 1). Reasons for not cryopreserving semen were azoospermia (*n*=5) and immotile sperm (*n*=2).

### Semen analysis after treatment

Of 115 patients who cryopreserved semen, 97 patients were alive and disease free, one patient was alive with disease, 16 patients had died and there was incomplete data for one patient when this analysis was performed. After treatment, 74 patients requested semen analysis at least once (median=2, range 1–7) and at varying times following treatment depending on personal circumstances. At the first analysis, a median interval of 2.9 years (range 0.1–12.3) after completion of chemotherapy, the majority were azoospermic (68 out of 74) and four patients were oligospermic (1×10^6^ per ml). Normal spermatogenesis was present in two patients (sperm concentrations of 25 million per ml and 50 million per ml), 79 and 54 months after treatment. Both patients had received only three of a planned eight cycles of MVPP. At subsequent analyses, recovery of spermatogenesis was detected in seven previously azoospermic patients although all remained severely oligospermic (sperm concentration <10×10^6^ per ml). Overall, a degree of recovery of spermatogenesis occurred in 13 out of 74 patients at a median interval of 5.1 years (range 3.3–13.1) after treatment. To our knowledge, four patients in whom spermatogenesis recovered have fathered children naturally.

### Assisted reproduction using cryopreserved semen

**Figure 3:**
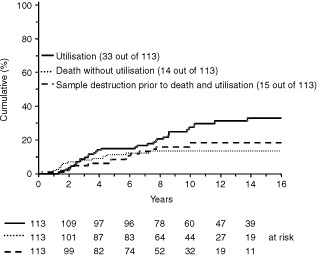
Cumulative rates of semen utilisation, death before utilisation and sample destruction without utilisation or death in patients with Hodgkin's disease (Kaplan–Meier method).

**Figure 4:**
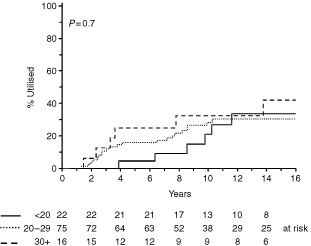
Cumulative rates of semen utilisation according to age at diagnosis of Hodgkin's disease (Kaplan–Meier method).

Data regarding utilisation was complete for 113 out of 115 patients who stored semen. The actuarial rate of utilisation 10 years from storage is 27%, the rate of destruction of semen without utilisation and before death of the patient is 19% and the rate of death without utilisation is 13% (Figure 3

). Cryopreserved samples from 29 patients have been destroyed due to death (*n*=15), recovery of spermatogenesis (*n*=4) or at patient request with no reason specified (*n*=10). Patients who remain alive who have neither used nor destroyed cryopreserved semen have been followed for a median of 10.1 years. At the time of analysis, cryopreserved semen had been utilised for assisted reproduction by 33 couples. Grouping of patients according to whether or not they already had children or marital status at diagnosis revealed no group of patients who were significantly more or less likely to attempt assisted reproduction using cryopreserved semen (data not shown). Similarly, analysis by age at diagnosis also showed no significant difference in utilisation after 10 years (Figure 4

) although numbers in each group were small.

During the course of this study a number of methods of assisted reproduction were used as treatments evolved and new techniques were introduced. At the start of the study in 1978, the only option was simple vaginal insemination or artificial insemination with husband's sperm (AIH). *In vitro* fertilisation (IVF) was offered from 1984 onwards, and intracytoplasmic sperm injection (ICSI) from 1995 (van Steirteghem *et al*, 1993). AIH was superceded by direct insemination into the uterine cavity (IUI) in 1995 (Horne *et al*, 1998). Of 33 couples receiving assisted reproduction, AIH was used by 28, IUI by two, and IVF or ICSI by three. Seven couples who originally received AIH went on to have IVF or ICSI as these techniques became available. In nine out of 33 couples, 10 pregnancies occurred of which eight resulted from AIH and two from IVF. One pregnancy (AIH) was terminated due to foetal malformation (hydrocephalus and cervical hygroma; normal female karyotype) but the remaining nine pregnancies resulted in the birth of 8 healthy singletons and one triplet of males to give a live birth rate of 27%. The triplet of males was the result of implantation of frozen thawed embryos from a second attempt at IVF using cryopreserved semen and ICSI, a particularly unusual case that has already been reported elsewhere (Horne *et al*, 2001). In 23 couples assisted reproduction was not successful (see Table 1

**Table 1:**
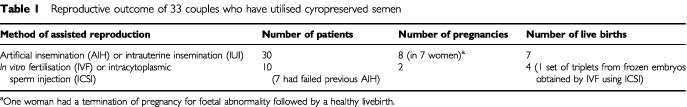
Reproductive outcome of 33 couples who have utilised cyropreserved semen

for summary of reproductive outcomes).

## DISCUSSION

The use of combination chemotherapy regimens has dramatically improved the prognosis for patients with Hodgkin's disease. However, gonadal toxicity is common and for a number of years patients have been routinely counselled regarding this important late effect of treatment and offered semen cryopreservation. This policy initially met with the concern that sperm quality was adversely affected by Hodgkin's disease itself and semen was therefore unlikely to be of use for cryopreservation and subsequent assisted reproduction (Whitehead *et al*, 1982). Several investigators have demonstrated relatively poor semen quality in patients with Hodgkin's disease compared to healthy controls (Redman *et al*, 1987; Shekarriz *et al*, 1995), and patients with non-Hodgkin's lymphomas (Botchan *et al*, 1997; Lass *et al*, 1998) although, consistent with the current study, a significant proportion of men with Hodgkin's disease are found to have semen quality within the normal range at diagnosis.

There have been dramatic advances in reproductive medicine since the first reports of successful artificial insemination using cryopreserved semen from patients with Hodgkin's disease (Scammell *et al*, 1985). The increasing availability of techniques such as ICSI means that semen containing even very low numbers of sperm should, nevertheless be cryopreserved (Lass *et al*, 1999). Furthermore, although in this series one pregnancy was terminated for foetal malformation there is no known increased risk of this complication in pregnancies resulting from the use of cryopreserved semen stored by patients with Hodgkin's disease or other malignancies (Lansac *et al*, 1997).

This study confirms the high rates of sterility previously reported for MVPP and ChlVPP/EVA hybrid chemotherapy (Whitehead *et al*, 1982; Clark *et al*, 1995) and highlights the increasing use of semen cryopreservation over time which probably reflects both a greater awareness of the importance of fertility issues for patients with a good prognosis and the availability of appropriate facilities. Although there are anecdotal reports of successful pregnancies following the use of cryopreserved semen in men with Hodgkin's disease (Scammell *et al*, 1985; Redman *et al*, 1987; Milligan *et al*, 1989), to our knowledge, there are no published data describing the proportion of long term survivors of Hodgkin's disease in the UK who attempt assisted reproduction and achieve success. Our retrospective data show that most patients have not used their cryopreserved semen (27% utilisation 10 years from storage, Figure 3) the reasons for which are unclear. Although factors such as disease status following treatment, age, marital status or number of children at diagnosis might be expected to have a bearing on reproductive decision making, our results implicate other, as yet unidentified factors. In the UK these may include lack of immediate access to assisted reproduction treatment as many health authorities do not currently provide NHS funding. The Regional IVF and Donor Insemination treatment centre at St Mary's Hospital in Manchester, for example, does provide NHS treatment but has a 3–4 year waiting list, does not treat couples who already have children living with them and has treated male factor infertility since only 1994. Workers from Australia (Audrins *et al*, 1999) have reported an even lower utilisation rate (7%) but in this study the mean follow-up time was only 3 years, a factor that may be important in evaluating this end point.

In the retrospective cohort reported here, approximately one-quarter have utilised cryopreserved semen following treatment for Hodgkin's disease and of these, approximately one-quarter have had a live birth. Vaginal insemination was the most commonly used method of assisted reproduction and the live birth rate of 27% for all methods used is consistent with, if not better than, expected outcomes for infertile couples (Human Fertilisation and Embryology Authority, 1999, annual report). However, pregnancy was not achieved in the majority of couples who wished to have a child and ways to improve reproductive potential in this group of patients including earlier and more frequent use of advanced reproductive techniques such as IVF and ICSI need to be considered. It should be remembered, however, that procedures more complex than artificial insemination are significantly invasive and women with normal fertility may find the demands made too stressful and/or time consuming. In addition, these techniques usually involve either a long waiting list or considerable expense if undertaken privately. Our finding that most patients have not yet used cryopreserved semen is intriguing and warrants further investigation. Do these men and their partners consider themselves still too young to start a family or have they opted never to have children? Is assisted reproduction acceptable to patients? Do they have access to all the reproductive technology currently available? And finally, are there fears regarding safety such as risk of disease transmission?

In addition to employing less toxic chemotherapy regimens wherever possible (Kulkarni *et al*, 1997) other strategies to overcome the impact of chemotherapy on reproductive function include the use of donor sperm for artificial insemination or IVF of the partner's eggs, hormonal manipulations to enhance recovery of spermatogenesis (Meistrich, 1998) and cryopreservation with subsequent autotransplantation of testicular germ cells (Brook *et al*, 2001). There is no doubt, however, that men about to embark on a programme of sterilising chemotherapy should be offered semen cryopreservation as routine in the knowledge that the live birth rate following utilisation is at least 25%, a figure that is set to rise as a consequence of steady advances in reproductive medicine.
